# Combined association of drinking water salinity and psychosocial distress with hypertension among reproductive-aged women in coastal Bangladesh: a population-based cross-sectional study

**DOI:** 10.1186/s12905-026-04771-x

**Published:** 2026-08-19

**Authors:** Ashraful Alam Siddique, Mahin Al Nahian

**Affiliations:** International Centre for Diarrhoeal Disease Research, Bangladesh (icddr,b), 68, Shaheed Tajuddin Ahmed Sarani Mohakhali, Dhaka, 1212 Bangladesh

**Keywords:** Bangladesh, Climate change, Drinking-water salinity, Hypertension, Psychosocial distress, Sodium, Women's health

## Abstract

**Background:**

Climate change-driven salinity intrusion into drinking water poses a structural risk for hypertension among women in coastal Bangladesh. Psychosocial distress from climate stressors may amplify this effect, but how these two factors interact jointly remains understudied.

**Methods:**

This population-based cross-sectional survey included 4,693 ever-married women aged ≥ 18 years in coastal Bangladesh, using probability proportionate to size sampling in high- and low-salinity areas. Hypertension was defined as SBP ≥ 140 mmHg, DBP ≥ 90 mmHg, or antihypertensive use. Psychosocial distress was measured by the PHQ-ADS composite (GAD-7 + PHQ-9; low: 0–19, moderate: 20–29, severe: ≥30). Drinking water sodium was quantified continuously (per 100 mg/L) and categorically (< 1000, 1000 2000, > 2000 mg/L). Multivariable logistic regression estimated adjusted odds ratios (AORs), controlling for age, education, wealth, salinity zone, disaster exposure, and income loss. Effect modification was tested via interaction terms and stratification.

**Results:**

Hypertension prevalence was 24.9%; moderate/severe distress affected 75.4%. Significant independent associations included moderate distress (AOR 1.86; 95% CI 1.51–2.28), severe distress (AOR 1.88; 95% CI 1.51–2.32), and sodium (AOR 1.10 per 100 mg/L; 95% CI 1.02–1.35). Significant multiplicative interaction occurred between high sodium (> 2000 mg/L) and severe distress (interaction AOR 1.29; 95% CI 1.01–1.56; *p* = 0.011), with stratified AOR 2.45 (95% CI 1.79–3.36) for combined exposure. Sensitivity analyses confirmed robustness (combined exposure AORs 1.95–3.10).

**Conclusions:**

Psychosocial distress modifies the hypertension risk from climate-driven drinking water salinity among coastal Bangladeshi women, suggesting combined association. Integrated mental health screening, psychosocial support, and salinity mitigation are urgently needed to drive hypertension prevention and climate-health adaptations.

## Background

Climate change represents one of the most pressing global health challenges of the 21st century, with low-lying coastal regions facing disproportionate risks due to sea-level rise, increased frequency and intensity of cyclones, flooding, and progressive salinity intrusion into freshwater sources [1]. Bangladesh is among the countries most vulnerable to climate change and sea-level rise (CCSLR)- associated impacts, particularly in its coastal districts, where millions of people are chronically exposed to environmental stressors such as saline drinking water, flooding, and waterlogging [2]. These climate-related stressors disrupt livelihoods, food security, and access to healthcare, thereby amplifying existing social and health vulnerabilities [2, 3]. Women of reproductive age (15–49 years) living in coastal Bangladesh are especially susceptible to the health impacts of climate change due to gendered social roles, caregiving responsibilities, and limited control over household resources and mobility [4, 5]. In these settings, women are often responsible for water collection and household management, thereby increasing their direct exposure to saline water and climate-induced hardships [5]. Such structural and gendered vulnerabilities position women at the intersection of environmental exposure and adverse health outcomes.

A growing body of evidence links climate-sensitive environmental exposures- particularly drinking-water salinity- to elevated blood pressure and increased hypertension risk [6, 7]. In coastal Bangladesh, saltwater intrusion into groundwater aquifers has substantially increased sodium concentrations in drinking water, resulting in involuntary sodium intake that exceeds recommended levels [8]. Epidemiological studies from coastal Bangladesh have consistently demonstrated positive associations between drinking-water salinity and systolic and diastolic blood pressure, as well as higher prevalence of hypertension among exposed populations [9]. Unlike dietary sodium intake, exposure to sodium through drinking water is largely involuntary and difficult to mitigate at the individual or household level, rendering it a structural environmental determinant of cardiovascular risk [7].

Concurrently, climate change imposes significant psychosocial burdens that adversely affect mental health. Climate-related shocks- including cyclones, flooding, salinity intrusion, and livelihood insecurity- are increasingly recognized as drivers of anxiety, depression, and chronic psychological distress, particularly in low- and middle-income countries [10, 11]. The Lancet Countdown on Climate Change and Health has identified mental health impacts as a critical yet under-explored dimension of climate-associated health burden [12]. In Bangladesh, repeated exposure to environmental uncertainty and cumulative climate stressors result in sustained psychological distress rather than short-term trauma responses [13]. Psychosocial distress is a proven risk factor for hypertension and cardiovascular disease [14]. Chronic anxiety and depression are known to activate neuroendocrine stress pathways, including dysregulation of the hypothalamic-pituitary-adrenal axis and sustained sympathetic nervous system activation, leading to vascular dysfunction and elevated blood pressure [15, 16]. Anxiety disorders, in particular, have been associated with increased hypertension risk and poorer cardiovascular outcomes [17]. In climate-vulnerable settings, these biological pathways may be intensified as environmental stressors act as persistent triggers for psychological distress.

Despite growing evidence linking drinking-water salinity with hypertension and psychosocial distress with cardiovascular risk, few population-based studies have examined whether psychosocial distress modifies the cardiovascular effects of climate-related environmental exposures, particularly among women in climate-vulnerable coastal regions. This study aimed to examine the independent and joint associations between drinking-water salinity and psychosocial distress with hypertension among ever‑married women aged ≥ 18 years in coastal Bangladesh. It was hypothesized that psychosocial distress modifies the association between salinity exposure and hypertension, such that women exposed to both high salinity and high distress would have a greater risk than those exposed to either factor alone.

## Methods

### Study settings, and design

The study was conducted in the coastal region in Bangladesh; the climatic hotspot has been increasingly affected by climate change and sea level rise-associated salinity intrusion [2]. High- and low-salinity areas were identified using Bangladesh Water Development Board [18] groundwater quality monitoring data, applying an electrical conductivity cut-off of 3,000 µS/cm [7]. This study was reported in accordance with the Strengthening the Reporting of Observational Studies in Epidemiology (STROBE) guidelines for observational studies [19].

### Selection of study areas and sampling

**Fig. 1:**
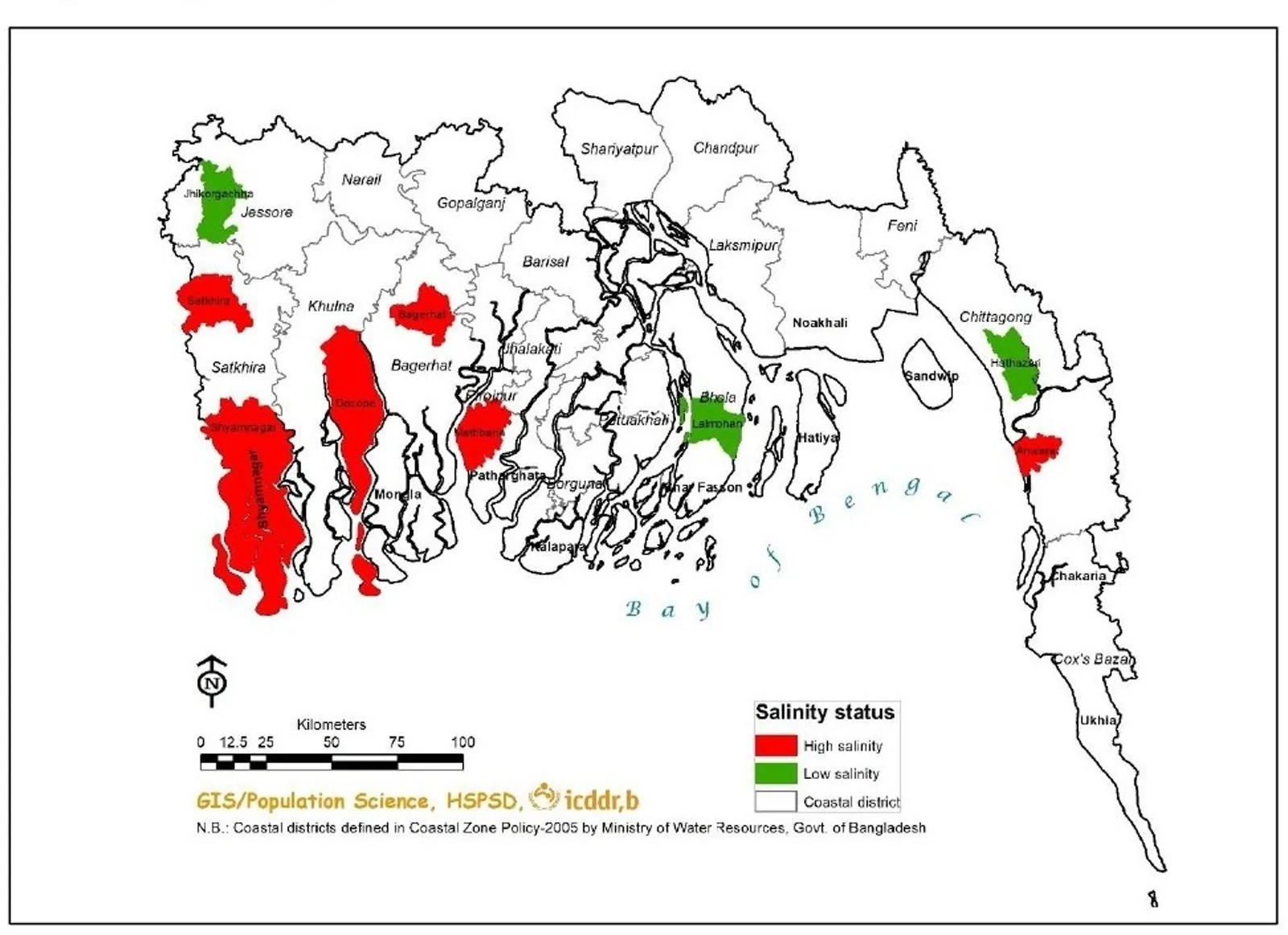
Study area maps

The study area included nine sub-districts (sub-districts) across seven coastal districts in three divisions, representing the entire coastal regime in Bangladesh [20]. The high-salinity study areas were, from Barisal division- (1) district: Pirojpur/ sub-district: Mathbaria; in Chattogram division (2) district: Chattogram/ sub-district: Anwara; in Khulna division, (3) district: Bagerhat/ sub-district: Bagerhat Sadar; (4) district: Khulna/ sub-district: Dacope; (5) district: Satkhira/ sub-district: Satkhira Sadar; and (6) sub-district: Shyamnagar. The low-salinity areas included, from Barisal division, (7) district: Bhola/ sub-district: Lalmohan; from Chattogram division, (8) district: Chattogram/ sub-division: Hathazari; and from Khulna division, (9) district: Jessore/ sub-district: Jhikargachha. A probability proportionate to size (PPS) sampling strategy was used, with a 2:1 ratio of high salinity to low salinity areas. Six high salinity and three low salinity sub-districts were selected for the study based on the groundwater salinity measurements (Fig. 1). Within each sub-district, 15 clusters (mouzas) were chosen using PPS. “Mouza” is the lowest administrative unit in Bangladesh, and the complete mouza list was collected from the Bangladesh Bureau of Statistics. The selection probability for each mouza was determined by dividing the number of households in a specific mouza by the total number of households in that sub-district. Though the outcome was varied, we rounded the Mouza/ cluster per sub-district to 15. We randomly selected 50 households in each cluster, keeping 20% additional for anticipated non-response, from the household listing. The sample size was calculated using an expected hypertension prevalence of 25%, a 95% confidence level, 1.5 design effect, and 20% anticipated non-response. This yielded a target sample of 6,750 households for the parent survey. Among interviewed women, 4,693 women aged ≥ 18 years with complete blood pressure and sodium exposure data were included in the present analysis.

### Participants

Eligible participants were ever-married women aged 15–49 years in the study. For blood pressure and hypertension analyses, women aged < 18 years were excluded due to age-specific norms, resulting in an analytical sample of 4,693 women (≥ 18 years). The study had information on all women respondents’ distress and drinking water salinity measurements, but carefully excluded the 15-17-year-old sub-group from analysis to ensure comparability with adult BP definitions. The household response rate was 94.8% (6,396/6,750), and the individual women’s response rate was 91.0% (6,092/6,698). The study analyzed data of 4,693 women in the 18–49 years range.

### Field implementation, data collection, and quality control (QC)

The National Institute of Population Research and Training (NIPORT) funded the household survey, and Associates for Community and Population Research (ACPR) implemented the survey by managing field activities and data collection. The study data collection team had fifty-four members, three 15-member data collection teams, three team supervisors, and six quality control officers. Before the field implementation of the study, the team underwent two weeks of extensive training in September-October 2023, with field practices on data collection, water sample collection, and blood pressure measurement [20]. The data collection was started from October 16, 2023.

A separate quality control (QC) checklist questionnaire was developed and administered. As part of the QC process, 5% of the sample was retested during the survey. We organized 2 re-cap sessions for the data collection team within the survey. The QC teams monitored overall field activities, including data collection, BP measurement, water sample collection process, etc. After the data collection was completed in December 2023, data cleaning started, and the master dataset was ready for analysis by April 2024. Data analysis was completed by September 2024. A study report was developed and shared with the NIPORT and the Ministry of Health and Family Welfare (MoHFW) stakeholders [20].

### Measures

#### Outcome variable 

Hypertension served as the primary binary outcome, defined as average systolic blood pressure (SBP) ≥ 140 mmHg or diastolic blood pressure (DBP) ≥ 90 mmHg, or current use of antihypertensive medication [21, 22]. Blood pressure was measured using validated automated devices (Omron HEM 7121 J) by trained staff, with the average of the second and third readings after ≥ 5 min rest in a seated position. Measurements were conducted under standardized resting conditions to reduce short-term variability.

#### Main exposure

The Generalized Anxiety Disorder-7 (GAD-7) and Patient Health Questionnaire-9 (PHQ-9) instruments have demonstrated reliability in Bangladeshi and South Asian contexts [23, 24]. Combining them captures co-occurring anxiety and depression common under climate-related stressors [25]; thus, we also adopted GAD-7 and PHQ-9 scales in this study. Women reported the frequency of anxiety symptoms (7 items) and depressive symptoms (9 items) over the past two weeks, preceding the survey. Responses were scored on a 4-point Likert scale (Never = 0 to Always = 3). Total scores were calculated separately for anxiety (range: 0–21) and depression (range: 0–27). To capture co-occurring symptoms, the validated Patient Health Questionnaire Anxiety Depression Scale (PHQ-ADS) composite was used by summing GAD-7 and PHQ-9 scores (range: 0–48), with categories: low distress (0–19), moderate distress [20–29], and severe distress (≥ 30) [24, 26].

#### Climate exposure

Drinking water samples were collected from the source or the respective household. We measured the salinity and sodium content in the drinking water samples by Horiba LAQUA twin Salt-11 and Horiba LAQUA twin Na-11- Pocket Meters, respectively, in a temporary set-up laboratory. Water sample sodium measures were examined continuously (per 100 mg/L increase) and categorically: <1000 mg/L (fresh/low), 1000–2000 mg/L (slight/moderate), > 2000 mg/L (high), based on thresholds used in previous studies conducted in coastal Bangladesh [7]. Household salinity zone was classified as low, moderate, or high using geographic/environmental data or self-report, capturing community-level intrusion separate from individual sodium intake [6].

#### Other climate vulnerability exposures

Binary variables (yes/no) included experience of ≥ 1 major climate change and natural disaster in the past 10 years and climate-related household income loss, reflecting acute and chronic stressors.

#### Covariates

Age (categorized: 18–24, 25–34, ≥ 35 years), education (none, primary [grades 1–5], secondary [grades 6–10], higher [≥ 11]), and household wealth quintile (asset based index derived from household assets and housing characteristics using principal component analysis) were included as confounders based on a priori literature review and conceptual frameworks of cardiovascular risk, including sociodemographic factors (age, education, wealth), environmental exposure (salinity zone), and climate-related stressors (disaster exposure and income loss) [20, 27–29]. All estimates used woman-specific survey weights.

### Statistical analysis

Descriptive statistics summarized sociodemographic characteristics, drinking water sodium concentrations, psychosocial distress, and hypertension prevalence. Continuous variables were reported as means (SD), and categorical variables as frequencies (%). Associations between sodium exposure, distress, and hypertension were examined using multivariable logistic regression, estimating adjusted odds ratios (AORs) with 95% CIs. Models controlled for age, wealth quintile, education, salinity zone, disaster exposure, and income loss. Effect modification was assessed hierarchically. First, we tested the multiplicative interaction term between high sodium exposure and severe psychosocial distress in an unadjusted logistic regression model. A statistical significance threshold of *p* < 0.05 (Wald test) was defined a priori. Second, given evidence of a statistically significant interaction, we conducted stratified analyses by sodium exposure categories, presenting both unadjusted and confounder-adjusted estimates. Analyses included women aged 18–49 with complete sodium data (*n* = 4,693). Sampling weights ensured representativeness, and robust standard errors accounted for household clustering. Statistical significance was set at *p* < 0.05. All analyses were conducted in Stata 18.5 (StataCorp, College Station, TX, USA).

## Results

### Sociodemographic, climate exposure, and health characteristics

A total of 4,693 women were included in the analysis. The mean age was 33.0 years (SD 8.7), with 44.1% aged ≥ 35 years. The mean years of formal education were 6.6 years, and nearly half of the women (47.9%) had secondary-level education. Household wealth was relatively evenly distributed across quintiles, although 22.1% belonged to the poorest group. Exposure to climate-related vulnerabilities was widespread. Over 42.8% of women were exposed to high drinking water sodium levels (> 2000 mg/L), and 27.3% lived in high salinity zones. More than three-quarters (76.3%) reported experiencing at least one major climate disaster in the past 10 years, while 58.3% reported climate-related household income loss. Psychosocial distress was common: 40.1% experienced moderate distress and 35.3% severe distress. The overall prevalence of measured hypertension was 24.9%, and 10.2% of women had co-occurring hypertension and moderate-to-severe psychosocial distress (Table 1).

**Table 1:** Sociodemographic and climate exposure characteristics of women (*n* = 4,693*) (descriptive statistics - frequencies and percentages)

Characteristics	Category	*n* (%) or Mean (SD)
Socio-demographics		
Age (years)	Mean age (SD)	**33.01 (8.7)**
	18 to 24 years	918 (19.57)
	25 to 34 years	1,706 (36.36)
	>=35 years	2,068 (44.07)
Education (women)	Mean education (SD, Range)	**6.56 (3.73**,** 0–18)**
	No education	518 (11.04)
	Primary	1,383 (29.46)
	Secondary	2,249 (47.92)
	Higher	543 (11.58)
Household wealth quintile	Poorest	1,035 (22.06)
	Poorer	1,062 (22.63)
	Middle	1,036 (22.08)
	Richer	931 (19.85)
	Richest	628 (13.38)
Climate vulnerability exposures		
Drinking water sodium (Na)	< 1000 mg/L	1603 (34.2)
	1000–2000 mg/L	1079 (23.0)
	> 2000 mg/L	2011 (42.8)
Salinity zone	Low	684 (14.58)
	Moderate	2,728 (58.12)
	High	1,281 (27.30)
Experienced ≥ 1 major climate disaster (past 10 yrs)	Yes	3,578 (76.25)
Household income loss (climate-related)	Yes	2,734 (58.27)
Health outcomes		
Psychosocial distress (gad-7/phq-9)	Low	1,157 (24.65)
	Moderate	1,881 (40.09)
	Severe	1,654 (35.26)
*†*Hypertension (measured)	Yes	1,169 (24.91)
Co-occurring conditions		
Both hypertension & moderate-severe distress	Yes	481 (10.24)

Percentages are based on weighted data and may not sum to exactly 100 due to rounding. Psychosocial distress categories were derived from validated GAD-7 and PHQ-9 instruments

*All estimates are weighted using the woman weight; rounded weighted counts are presented

†Hypertension is defined as average systolic blood pressure ≥ 140 mmHg or diastolic blood pressure ≥ 90 mmHg, or current use of antihypertensive medication

### Prevalence of hypertension by psychosocial distress and climate exposures

Hypertension prevalence increased markedly with worsening psychosocial distress, rising from 13.9% among women without distress to 28.0% among those with moderate distress and 29.1% among those with severe distress. Hypertension prevalence was also higher among women exposed to high drinking water sodium (> 2000 mg/L) compared to those exposed to lower concentrations (Table 2).

**Table 2:** Prevalence of hypertension by psychosocial distress and climate exposure (Bivariate analysis showing unadjusted associations)

Exposure variable	Category	Hypertension % (*n*/*N*)	95% CI
Psychosocial distress level	Low	13.9 (161/1,157)	11.9–15.9
	Moderate	28.0 (527/1,881)	26.0–30.0
	Severe	29.1 (481/1,654)	27.0-31.2
Drinking water sodium (Na)	< 1000 mg/L	24.4 (391/1,603)	22.3–26.5
	1000–2000 mg/L	23.4 (253/1,079)	21.0-25.8
	> 2000 mg/L	26.1 (525/2,011)	24.2–28.0
Salinity zone	Low	22.6 (155/684)	19.4–25.8
	Moderate	24.8 (678/2,728)	23.1–26.5
	High	26.3 (337/1,281)	23.8–28.8

Unadjusted prevalence ratios or percentages with 95% CIs

### Multivariable associations with hypertension

After adjustment for sociodemographic and climate-related covariates, both psychosocial distress and drinking water sodium exposure remained independently associated with hypertension. Compared with women without psychosocial distress, the odds of hypertension were significantly higher among those with moderate distress (AOR 1.86; 95% CI: 1.51–2.28) and severe distress (AOR 1.88; 95% CI: 1.51–2.32). Drinking water sodium concentration, modeled as a continuous variable, was positively associated with hypertension, with a 10% increase in adjusted odds of hypertension per 100 mg/L increase in sodium concentration (AOR 1.10; 95% CI: 1.02–1.35) (Table 3).

**Table 3:** Adjusted associations of psychosocial distress and climate exposures with hypertension (multivariable logistic regression, outcome: hypertension)

Predictor	Category	Adjusted Odds Ratio (aOR)	95% CI	*p*-value
Psychosocial distress (ref: Low)	Moderate	1.86	1.51–2.28	< 0.001
	Severe	1.88	1.51–2.32	< 0.001
Water sodium (Na) (per 100 mg/l increase)	Continuous	1.10	1.02–1.35	0.019

The model is adjusted for: age, wealth, Education, Salinity Zone, Disaster Exposure, and Income Loss

### Effect modification

The formal interaction test (Table 4) confirmed a statistically significant multiplicative interaction between high sodium exposure and severe psychosocial distress in the unadjusted model (interaction OR 1.45; 95% CI: 1.20–1.75; *p* < 0.001). This interaction remained significant after adjustment for confounders (interaction AOR 1.29; 95% CI: 1.01–1.56; *p* = 0.011). Stratified analyses demonstrated evidence of effect modification between psychosocial distress and sodium exposure. Within the high sodium exposure group (> 2000 mg/L), women with moderate distress had 1.65 times higher odds of hypertension (95% CI: 1.20–2.28), while those with severe distress had 2.45 times higher odds (95% CI: 1.79–3.36), compared to women without distress. On the other hand, the association between psychosocial distress and hypertension was weaker and less consistent in the moderate sodium exposure group (Table 5).

**Table 4:** Formal interaction tests for high sodium and severe psychosocial distress

Model	Interaction term	Estimate	95% CI	*p*-value
Unadjusted model	High sodium × Severe distress	OR 1.45	1.20–1.75	< 0.001
Adjusted model*	High sodium × Severe distress	AOR 1.29	1.01–1.56	0.011

*Adjusted for age, wealth, education, salinity zone, disaster exposure, and income loss

**Table 5:** Stratified analysis: association between psychosocial distress and hypertension by sodium exposure

Sodium (Na) exposure	Psychosocial distress	*n*	Hypertension % (*n*)	OR (95% CI)	AOR* (95% CI)	*p*-value^†^
Low sodium (< 1000 mg/L)	Low	356	12.9 (46)	1.00	1.00	--
	Moderate	707	24.2 (171)	2.14 (1.49–3.07)	1.73 (1.17–2.47)	0.005
	Severe Distress	540	32.2 (174)	3.20 (2.22–4.61)	2.43 (1.65–3.57)	< 0.001
Moderate sodium (1000–2000 mg/L)	Low	274	14.7 (40)	1.00	1.00	--
	Moderate	391	24.4 (96)	1.87 (1.24–2.82)	1.36 (0.87–2.14)	0.172
	Severe Distress	412	28.3 (117)	2.29 (1.52–3.45)	1.50 (0.94–2.37)	0.083
High sodium (> 2000 mg/L)	Low	526	14.2 (75)	1.00	1.00	--
	Moderate	783	27.0 (190)	2.23 (1.66-3.00)	1.65 (1.20–2.28)	0.002
	Severe Distress	702	33.2 (260)	3.00 (2.25-4.00)	2.45 (1.79–3.36)	< 0.001

*Adjusted for age, wealth, education, salinity zone, disaster exposure, and income loss

^**†**^p-value for adjusted model

### Associations with systolic blood pressure

Linear regression analyses examining systolic blood pressure as a continuous outcome showed that each incremental increase in psychosocial distress category was associated with a 0.83 mmHg increase in systolic blood pressure (95% CI: 0.06–1.60). Drinking water sodium was also positively associated with systolic blood pressure, increasing by 0.64 mmHg per 100 mg/L increase (95% CI: 0.39–0.90). The interaction term between psychosocial distress and sodium exposure from saline drinking water sources was negative and did not reach statistical significance (β -0.18; *p* = 0.077), suggesting limited evidence of interaction on the continuous blood pressure scale (Table 6).

**Table 6:** Associations of psychosocial distress and sodium exposure with systolic blood pressure (linear regression)

Predictor	β-coefficient (mmHg)	95% CI	*p*-value
Psychosocial distress (per category increase)	0.83	0.06–1.60	0.034
Water sodium (Na) (per 100 mg/L increase)	0.64	0.39–0.90	< 0.001
Interaction term: Distress × Sodium	-0.18	-0.38-0.02	0.077

Adjusted for age, wealth, education, salinity zone, disaster exposure, and income loss

### Sensitivity analyses

Sensitivity analyses confirmed the robustness of findings. The interaction remained significant across alternative sodium thresholds and exclusions (*p* < 0.05), with combined exposure odds ratios ranging from 1.95 to 3.10 (all *p* < 0.001), indicating consistently elevated hypertension risk when both high drinking water sodium and severe distress co-occur (Table 7).

**Table 7:** Sensitivity analyses for the multiplicative interaction and combined exposure effects on hypertension

Sensitivity analysis	High sodium definition / Distress grouping	Interaction aOR (95% CI)	*p*-value	Combined exposure aOR (High Na + Severe distress vs. Low Na + Low distress)	*p*-value (Combined)
Main analysis	> 2000 mg/L (high) × Severe (≥ 30)	1.29 (1.01–1.56)	0.011	2.45 (1.79–3.36)	< 0.001
Sodium (Na) cut-off	> 1000 mg/L (Low)× Severe	1.35 (1.08–1.68)	0.007	2.62 (1.92–3.58)	< 0.001
Distress grouping	Moderate/Severe combined (≥ 20) vs. Low (0–19)	1.18 (0.98–1.42)	0.078	1.95 (1.48–2.57)	< 0.001
Exclude medication users	> 2000 mg/L × Severe (measured BP only)	1.41 (1.12–1.78)	0.004	2.78 (1.98–3.90)	< 0.001
Unadjusted model	> 2000 mg/L × Severe	1.45 (1.20–1.75)	< 0.001	3.10 (2.35–4.09)	< 0.001

## Discussion

This population-based study provides novel evidence of significant interaction between climate-driven drinking-water sodium and psychosocial distress in elevating hypertension risk among women in coastal Bangladesh. The findings highlight intersecting burdens of climate vulnerability and non-communicable disease in this high-risk setting, where there-quarters of women experience moderate-to-severe distress The study extends emerging literature showing that climate change disproportionately impacts women’s health via linked environmental, psychosocial, and physiological pathways [30], particularly in salinity-affected South Asian coastal zones.

The independent association between psychosocial distress and hypertension aligns with established neuroendocrine mechanisms. The independent association between psychosocial distress and hypertension aligns with established neuroendocrine mechanisms. Chronic anxiety and depression activate the hypothalamic-pituitary-adrenal axis and sustain sympathetic nervous system activation, leading to vascular dysfunction and elevated blood pressure [15–17, 31].

In climate-vulnerable contexts like coastal Bangladesh, repeated exposure to cyclones, flooding, salinity intrusion, and livelihood insecurity likely serves as a source of chronic, cumulative stress rather than discrete traumatic events. Although the cross-sectional design precludes causal inference, the robust associations persisting after extensive covariate adjustment support psychosocial distress as a plausible chronic pathway from climate vulnerability to cardiovascular outcomes. These findings align with evidence identifying climate change as an upstream determinant of mental health outcomes, particularly anxiety and depressive disorders, in low- and middle-income countries [25, 32].

Drinking-water sodium represents an involuntary, structural environmental exposure in this setting, unlike dietary sodium which individuals can partially regulate through behavioral choices. The observed dose-response relationship extends prior evidence from coastal Bangladesh, positioning drinking water sodium as a structural environmental driver of cardiovascular disease [7–9].

This study’s key contribution is the demonstration of effect modification between severe psychosocial distress and high drinking-water sodium on hypertension risk. This pattern is consistent with syndemic theory, wherein co-occurring psychosocial and environmental adversities amplify disease risk beyond additive effects [29, 33]. Biologically, chronic stress may heighten salt sensitivity via altered renal sodium handling, inflammation, and vascular dysregulation [16], while socially, distress may impair adaptive coping and resource access, exacerbating environmental vulnerability [28]. Notably, the persistence of associations after adjustment for disaster experience and income loss suggests that chronic, cumulative climate stressors may be more influential than discrete extreme events in shaping long-term cardiovascular risk [34].

The socioeconomic gradients observed support structural vulnerability frameworks emphasizing that social position shapes both exposure and resilience to health risks [35, 36]. However, the association between distress and hypertension was not fully explained by wealth, indicating that psychosocial pathways operate across socioeconomic strata.

The study has important implications for climate-water-health adaptation and health policy reformation. Mental health screening and psychosocial support should be integrated into primary care and non-communicable disease prevention programs in climatic hotspots, consistent with WHO recommendations [37]. Climate-vulnerable communities, exposed to high salinity from drinking water sources, should be provided with a fresh water supply; interventions to manage drinking-water salinity- such as rainwater harvesting, managed aquifer recharge, and community-scale desalination, and other alternative options should be recognized as public health interventions with potential cardiovascular benefits [9, 38]. Our findings suggest that such interventions may yield particularly large benefits for women experiencing high psychosocial stress.

Sensitivity analyses confirmed the robustness of the findings. The multiplicative interaction between high drinking-water sodium and severe psychosocial distress remained statistically significant across most alternative specifications, and combined exposure effects were consistently elevated, reinforcing the substantial joint cardiovascular burden of chronic salinity exposure and severe distress in this climate-vulnerable population [16, 39].

### Strengths and limitations

This study has several strengths, including a large population-based sample, high response rates (95% household, 91% women), use of validated instruments (GAD-7, PHQ-9), objective measurement of blood pressure and drinking water sodium, and rigorous sampling procedures. However, we also acknowledge several limitations of the study. First, we only measured the salinity and sodium concentration value with Horiba Pocket Meters, and do not have the concentration of other dissolved minerals in the samples. Such effort needed lab testing of all the samples, with substantial expenses, which were beyond the means of our limited study funding. Second, drinking water samples were collected in the post-monsoon season, when salinity concentration values are usually low; furthermore, the sample measurement was conducted at a single time point, potentially underestimating long-term variability due to seasonal salinity fluctuations in drinking water sources. Third, psychosocial distress was measured over the past two weeks, which may not fully reflect chronic exposure to cumulative climate stressors. Fourth, the cross-sectional design limits causal inference, as temporality is unclear. Fifth, unmeasured confounding factors such as body mass index (BMI) and diabetes could not be addressed; these were not collected in the parent study and should be included in future research. Sixth, the PHQ-ADS composite may capture overlapping symptoms of anxiety and depression, potentially inflating distress estimates; however, the use of validated cut-offs partially addresses this concern.

## Conclusions

The large population-based study from a highly climate-vulnerable South Asian setting provides novel evidence that psychosocial distress- a frequently overlooked dimension of climate change impacts- significantly modifies the association between climate-driven drinking-water sodium and hypertension risk among women of reproductive age in coastal Bangladesh. The observed joint effect underscores that the cardiovascular burden of climate change cannot be adequately addressed by examining environmental or psychosocial pathways in isolation.

The authors recommend a paradigm shift in public health policy and health adaptation practice toward integrated, multisectoral approaches: (1) incorporating routine mental health screening and community-based psychosocial support into primary healthcare and hypertension management programs in coastal and climate-vulnerable regions; (2) prioritizing gender-responsive structural interventions, such as climate-adapted water supply solutions, as essential public health measures to reduce a major environmental driver of cardiovascular disease; and (3) adopting syndemic-informed frameworks in research, policy, and programming to simultaneously address the interconnected burdens of climate-related psychosocial distress, environmental exposures, and non-communicable diseases rooted in shared structural vulnerabilities. This warrants a longitudinal study to clarify the temporal and causal potential mechanism of the climate–mental health–cardiovascular nexus within the framework of a climate-resilient health system to facilitate context-specific health adaptations in Bangladesh and beyond.

## Data Availability

The datasets generated and/or analysed during the current study are not publicly available due to institutional data policies but are available from the corresponding author on reasonable request.

## Abbreviations

%: Percentage
AOR: Adjusted Odds Ratio
BP: Blood Pressure
BWDB: Bangladesh Water Development Board
CI: Confidence Interval
CVD: Cardiovascular disease
DBP: Diastolic Blood Pressure
GAD: 7–Generalized Anxiety Disorder 7
PHQ: 9–Patient Health Questionnaire 9
PHQ: ADS–Patient Health Questionnaire Anxiety Depression Scale
PPS: Probability Proportionate to Size
SBP: Systolic Blood Pressure
SD: Standard deviation
WHO: World Health Organization
